# Encouraging COVID-19 vaccination by focusing on anticipated affect: A scoping review

**DOI:** 10.1016/j.heliyon.2023.e22655

**Published:** 2023-11-18

**Authors:** Tsuyoshi Okuhara, Ritsuko Shirabe, Yumi Kagawa, Hiroko Okada, Takahiro Kiuchi

**Affiliations:** a Department of Health Communication, School of Public Health, Graduate School of Medicine, The University of Tokyo, Tokyo, Japan; b Department of Health Communication, Graduate School of Medicine, The University of Tokyo, Tokyo, Japan

**Keywords:** COVID-19, Pandemic, Anticipated affect, Emotion, Vaccine hesitancy, Vaccination, Immunization, Vaccines, Health communication

## Abstract

**Objective:**

This study reviewed studies of the anticipated affect related with COVID-19 vaccination to understand gaps in currently available studies and practice implications.

**Methods:**

We systematically searched MEDLINE, CINAHL, and other multiple databases for English language articles of studies that investigated COVID-19 vaccination related anticipated affects.

**Results:**

We identified seventeen studies. Thirteen studies focused anticipated regret from inaction (i.e., not vaccinated). Other studies focused anticipated regret from action (i.e., vaccinated), guilt from inaction, pride from action, and positive feelings from action. Eleven studies showed that anticipated regret from inaction was significantly associated with COVID-19 vaccination behavior or intention. Three of the 11 studies showed that anticipated regret from inaction was more strongly associated with vaccination behavior or intention than cognitive belief.

**Conclusion:**

Most studies showed that positive associations between anticipated regret and COVID-19 vaccination outcomes. The use of messages that target cognitive beliefs as well as those that appeal to anticipated affect may be effective to promote COVID-19 vaccination. However, most studies employed a cross-sectional design and examined negative affect. Future studies should adopt an experimental design as well as examine positive affect.

## Highlights

• We reviewed studies on anticipated affect associated with COVID-19 vaccination.
• A total of 17 studies were identified in this scoping review.
• Most studies examined anticipated regret from inaction (i.e., not vaccinated).
• Anticipated regret was significantly associated with vaccination in most studies.
• Anticipated regret may be equally or more important than cognitive beliefs.

## 1 Introduction

Millions of infections and hundreds of thousands of deaths have been caused by the coronavirus disease 2019 (COVID-19) pandemic worldwide [1]. Vaccines against COVID-19 were developed with unprecedented rapidity. The United Kingdom, the United States, and Israel first offered the COVID-19 vaccination in December 2020 [[2], [3], [4]]. Since then, high efficacy of COVID-19 vaccination has been reported worldwide [5]. Nearly 2 years after the COVID-19 vaccination program began worldwide, billions of people have safely received the COVID-19 vaccine. However, a surprisingly large number of people are still hesitant to receive the COVID-19 vaccine [6]. Additionally, regular multiple doses are recommended because the effect of the COVID-19 vaccines declines over a period of several months [7,8]. COVID-19 vaccination is crucial even in the age of living with COVID-19. Health professionals need to disseminate messages continuously to promote COVID-19 vaccination.

Cognitive behavioral models have been focused on communication strategies to promote vaccination. (e.g., theory of planned behavior) [9,10]. Studies on COVID-19 vaccination communication have also emphasize cognitive beliefs, such as perceived vaccine efficacy and safety [[11], [12], [13]]. Cognitive behavioral models trust individuals’ rationality and assume that cognitive beliefs, by which individuals weigh costs and benefits, predict future vaccination behavior [14]. However, such cognitive appeals have been criticized for their bias towards cognitive influences on health behavior and their neglect of affective influences [15].

In recent years, studies have paid attention to the affective determinants of health behavior as complements to cognitive determinants [16]. Anticipated affect (i.e., the expectation of one's affective response to the target behavior) has received the most attention. Most studies have focused on the anticipated negative affect from inaction, such as anticipated regret from inaction [17]. In the case of COVID-19 vaccination, if people do not get vaccinated, they may anticipate a feeling of regret or guilt if they are ever infected or when they transmit COVID-19, thus choosing to receive the vaccination to avoid feeling the negative affect. Several studies have shown that anticipated regret from inaction is related with adopting health behaviors, such as physical activity [18], cancer screening [19,20] and vaccination [21]. Studies have found that anticipated regret from inaction more strongly predicts HPV [22] and influenza vaccination [23] than cognitive appeals.

Additionally, government agencies and health professionals may also be able to appeal to anticipated positive affect from action, such as pride, to encourage COVID-19 vaccination (e.g., “Be proud to vaccinate against COVID-19 to protect your loved ones.”). In contrast, anticipated negative affect (e.g., regret) from action may discourage the receipt of the COVID-19 vaccine; for example, Some may choose not to vaccinate against COVID-19 because they presume to regret having vaccinated against it if they suffer from adverse events.

Thus, anticipated affect may be related and have an impact on COVID-19 vaccination. A review of previous studies of anticipated regret on the association and impact on COVID-19 vaccination will help create effective message strategies to promote COVID-19 vaccination. Our scoping review aimed to overview studies that have investigated anticipated affects to promote COVID-19 vaccination. We specifically aimed to examine the affective versus the cognitive impact on COVID-19 vaccination.

RQ1 What is known from previous studies regarding the anticipated affect associated with COVID-19 vaccination? (e.g., study characteristics).

RQ2 What are the gaps in the studies to date on anticipated affect associated with the COVID-19 vaccination, and what should been to fill those gaps?

RQ3 What types of anticipated affect (versus cognitive beliefs) are associated with and influence COVID-19 vaccination?

RQ4 Can evidence-based recommendations be made on communication focusing on anticipated affect to promote COVID-19 vaccination?

## 2 Methods

This review was conducted by complying with the Preferred Reporting Items for Systematic Reviews and Meta-Analyses Extension for Scoping Reviews (PRISMA-ScR) [24].

### 2.1 Literature search

The following databases were searched: MEDLINE, Cumulative Index to Nursing and Allied Health Literature (CINAHL), PsycINFO, PsycARTICLES, Academic Search Complete, Scopus and Web of Science. Searches were conducted by the first author (TO) on July 18, 2022. In these searches, the publication year was filtered to 2019 onward. The following combinations of keywords regarding anticipated affect and vaccination were used to search for abstracts (see Appendix 1 for the precise of keywords). We used Rayyan QCRI software for systematic and comprehensive search and documentation of the selection process [25]. Manual searches for the reference lists of the literatures was conducted to find potentially eligible studies. We also used Google Scholar to warrant triangulation of the study selection.

### 2.2 Eligibility criteria

This review included quantitative or qualitative studies that investigated the COVID-19 vaccination relate anticipated affects. Studies on COVID-19 vaccination were included; literatures of other vaccines were excluded (e.g., HPV and MMR). We included any type of anticipated affect. All study designs were included such as quantitative studies, qualitative studies, and review studies. Literatures of any quantitative outcomes such as behavior and intention were included. Regarding participants characteristics, studies were included for all ages, genders, ethnicities, or countries. Literatures written in other languages than English were excluded. Literatures not published as full-text articles were excluded. Gray literature (e.g., dissertations) was eligible when its eligibility was confirmed with sufficient information.

### 2.3 Study selection

**Fig. 1:**
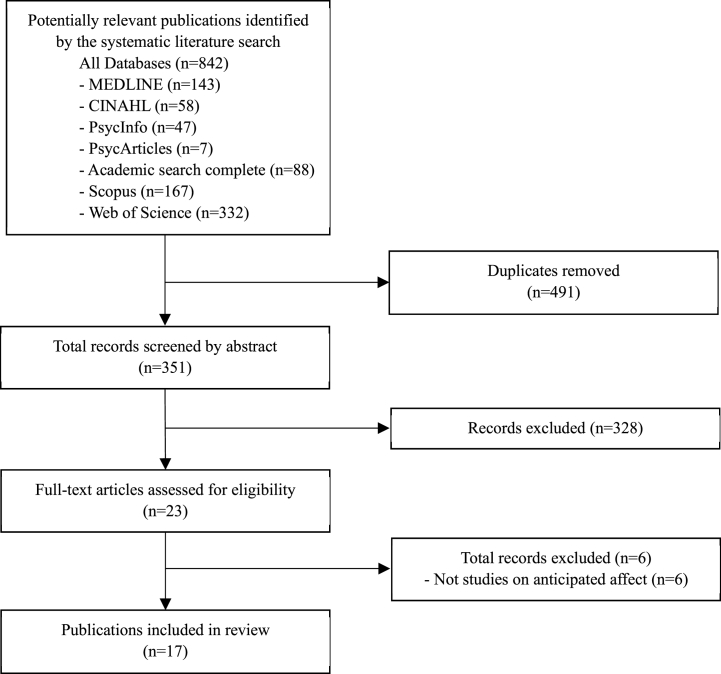
Search process flow chart.

We used the Rayyan QCRI software [25] for study selection. The first author (TO) and third author (YK) screened the titles and abstracts of all the studies that met the eligibility criteria. Then, the first author (TO) and second author (RS) screened independently the full-text versions of potentially relevant studies. Two reviewers discussed in the case of disagreements until they came to an agreement. In the case of disagreement, the third reviewer (the fourth author, HO) was invited for additional discussion. Fig. 1 shows the flow of the literature search and screening.

### 2.4 Data extraction and synthesis

The first author (TO) performed data extraction using a customized data extraction form, and the second author (RS) confirmed the reliability of the extracted data with reference to the full texts of the literatures. The first author (TO) and the second author (RS) discussed disagreements until they came to an agreement. The following data were extracted: publication type, study characteristics, study aim, study design, type of anticipated affect, type of cognitive beliefs (when described), study setting, participant characteristics, methodology, key findings, and mediating models (when described). The basic information of the included literatures were numerically summarized. Results of this review were synthesized and described in a narrative way to answer the research questions.

## 3 Results

### 3.1 Study characteristics

We identified seventeen studies (Table 1), of which, seven studies were published in 2021, and 10 studies were published in 2022. Three studies were conducted in the United States of America; three in China; two in the United Kingdom; one each in Norway, Ireland, Italy, Australia, Israel, Pakistan, Bangladesh, and Nigeria, and one in multiple countries across the globe. Fourteen studies examined anticipated regret from inaction, three examined anticipated regret from action, two examined anticipated guilt from inaction, two examined anticipated pride from action, and one examined anticipated positive feelings from action. Sixteen were quantitative studies that examined the participants' own vaccinations, and one was a qualitative study that examined mothers’ perceptions of vaccinating their children. 15 were quantitative cross-sectional studies, and one was an intervention study. The minimum, maximum, and median number of participants was 162, 3039, and 841, respectively, for the quantitative studies. 12 studies measured intention to receive vaccination, three studies assessed vaccination behavior (self-reported), and one study assessed vaccine hesitancy. All quantitative studies examined cognitive beliefs (e.g., perceived susceptibility of infection) of COVID-19 and anticipated affect.

**Table 1:** Summary of included studies.

Author (year)	Country	Anticipated affect	Design	Participants (n)	Primary outcome	Key findings^a^	Mediating model
Rosental et al. (2021) [26]	Israel	Inaction regret	Cross-sectional	Nursing and medical students (628)	Intention	Benefits of vaccine (β = .26), barriers (β = − .15), attitude (β = .21), inaction regret (β = .17), self-efficacy (β = .13), cues to action (β = .07), and susceptibility (β = .06) were significantly associated with intention. Severity, subjective norms and general health motivation were not significantly associated with intention.	None
Rountree et al. (2021) [27]	Ireland	Inaction regret	Cross-sectional	Adults (1995)	Intention	Inaction regret (β = .29), instrumental attitudes (β = .25), barriers (β = - .23), subjective norms (β = .11), affective attitudes (β = .08), efficacy expectations (β = .07), self-efficacy (β = - .04) were significantly associated with intention. Susceptibility, severity, cues to action, perceived control, social norms were not significantly associated with intention.	None
Radic et al. (2021) [41]	Regions across the globe	Action pride, inaction guilt	Cross-sectional	International adult travelers (1221)	Intention	Action pride (β = .938) and inaction guilt (β = .089) were significantly associated with personal norm which directly predicted intention (β = .943).	Pride and guilt predicted intention, mediated by personal norm.
Hossain et al. (2021) [39]	Bangladesh	Inaction regret	Cross-sectional	Adults (1497)	Hesitancy	Among the theory of planned behavior constructs, subjective norm (β = - .31), attitude (β = .27), inaction regret (β = - .18), behavioral control (β = - .05) were significantly associated with hesitancy.	None
Wolff (2021) [28]	Norway	Inaction regret, action regret	Cross-sectional	Adults (1003)	Intention	Attitude (β = .30), inaction regret (β = .22), social norm (β = .17), action regret (β = - .14), and perceived control (β = .08) were significantly associated with intention. Susceptibility and seriousness were not significantly associated with intention.	None
Goffe et al. (2021) [29] b	UK	Inaction regret	Cross-sectional	Adults (1660)	Intention	Attitude (β = .227), trust in the government (β = .205), subjective norms (β = .193), inaction regret (β = .170), benefits of vaccine (β = .149), and safety of vaccine (β = .05) were significantly associated with intention. Susceptibility, severity, and perceived control were not significantly associated with intention.	None
Capasso et al. (2021) [35]	Italy	Action pride, inaction regret	Intervention (between subject design)	Adults (484)	Intention	Intention was significantly higher in the cognitive attitude plus action pride message condition (M = 4.06; SD = 1.12) compared to the control group (M = 3.56; SD = 1.33). In contrast, no significant differences were found between cognitive attitude message or cognitive attitude plus inaction regret message and control conditions.	The effect on intention was fully mediated by cognitive attitude and action pride.
Antonopoulou et al. (2022) [30] b	UK	Inaction regret	Cross-sectional	A representative sample (1660)	Intention	Attitude (β = .242), subjective norm (β = .190), inaction regret (β = .188), trust to authorities for vaccine approval (β = .171), vaccine benefits (β = .128), vaccine safety (β = .069), knowledge (β = .047) were significantly associated with intention.	None
Wang (2022) [40]	China	Inaction guilt	Cross-sectional	Adults (460)	Intention	Benefits of vaccine (β = .39), susceptibility (β = .27), hope (β = .29), self-efficacy (β = .17), and inaction guilt (β = 0.11) were significantly associated intention. Severity was not significantly associated with intention.	Susceptibility and benefits of vaccine were associated with intention, partially mediated by inaction guilt.
Khayyam et al. (2022) [36]	Pakistan	Inaction regret	Cross-sectional	Healthcare works (680)	Behavior (self-reported)	Susceptibility was significantly associated with inaction regret (β = .59). Inaction regret (β = .46), attitude (β = .25), behavioral control (β = .23), and subjective norm (β = .12) were significantly associated with behavior.	Susceptibility was associated with behavior, mediated by inaction regret.
She et al. (2022) [31]	China	Inaction regret, action positive feelings	Cross-sectional	Doctors (362) and nurses (1702)	Intention	Subjective norm showed the significant strongest association with intention. Likelihood of protecting, inaction regret, action positive feelings, self-efficacy and social norms were also significantly associated with intention.	None
Schuster et al. (2022) [48]	Australia	Inaction guilt	Qualitative	Mothers (30)	Not applicable	Participants described anticipated guilt from failing to meet societal expectations around being a ‘good mother’ within an intensive mothering ideology either by accepting or rejecting COVID-19 vaccination for their children.	None
Short et al. (2022) [32]	US	Inaction regret	Cross-sectional	Young adults (526)	Intention	Inaction regret (β = .34), attitudes (β = .26), agreement (β = .22), and subjective norms (β = .12) were significantly associated with intention. Susceptibility, severity, and barriers were not significantly associated with intention.	None
Erubami et al. (2022) [38]	Nigeria	Inaction regret, action regret	Cross-sectional	Social media users (436)	Behavior (self-reported)	Severity of vaccine adverse events (β = - .51), barrier (β = - .47), susceptibility of vaccine adverse events (β = - .44), action regret (β = - .42), fear of vaccine (β = - .38) were significantly associated with behavior. Benefits of vaccine and inaction regret were not significantly associated with behavior.	None
Liu et al. (2022) [33]	China	Inaction regret	Cross-sectional	Adults (438)	Intention	The more participants seek or discuss COVID-19 vaccine-related information, the more likely they would show greater anticipated inaction regret and perceive higher levels of collective responsibility which, in turn, result in stronger COVID-19 vaccination intention.	Vaccine information seeking and discussing were associated with vaccination intention, mediated by anticipated regret and collective responsibility.
Hayashi et al. (2022) [34]	US	Inaction regret	Cross-sectional	Adults (162)	Intention	Inaction regret was not significantly associated with intention. Behavioral control (β = .29), attitude (β = .23), and community benefit (β = .23) were significantly associated with intention.	None
Huang et al. (2022) [37]	US	Inaction regret, action regret	Cross-sectional	Primary care professionals (1047)	Behavior (self-reported)	Vaccine confidence (OR = 4.28), inaction regret (OR = 1.69), susceptibility (OR = 1.29), social norms (OR = 1.05) and action regret (OR = .52) were significantly associated with behavior.	None

a Key findings described psychosocial variables; variables that cannot be changed such as socio-demographics and past vaccination behaviors were not described in the table.

b These two studies analyzed the same data using different models.

### 3.2 Summary of key findings

Table 2 shows the summary of the results. Most studies (nine studies) investigated the relationship between anticipated inaction regret and intention to receive vaccination by adopting a cross-sectional design. Among those nine studies, eight reported that anticipated regret from inaction was significantly and positively associated with intention [[26], [27], [28], [29], [30], [31], [32], [33]], and two out of the eight studies reported that anticipated regret from inaction was significantly, positively, and more strongly associated with vaccination intention than cognitive beliefs [27,32]; however, one cross-sectional study reported no significant association [34]. Additionally, one intervention study showed that anticipated regret from inaction had no significant impact on intention to receive vaccination [35].

**Table 2:** **Summary of key findings of quantitative studies by type of anticipated affect and primary outcome** *.

	Inaction regret	Action regret	Inaction guilt	Action pride	Action positive feelings
Behavior (self-reported)	CM◎ [36], C● [38], C〇 [37]	C⊖ [38], C⊖ [37]			
Intention	C〇 [26], C◎ [27], C〇 [28], C〇 [29], IM● [35], C〇 [30], C〇 [31], C◎ [32], CM〇 [33], C● [34]	C⊖ [28]	CM〇 [41], CM〇 [40]	CM〇 [41], IM〇 [35]	C〇 [31]
Hesitancy	C⊖ [39]				

* When multiple outcomes were measured, primary outcomes (e.g., vaccination intention) were focused on this table.

C: Cross-sectional study.

I: Intervention study.

CM: Cross-sectional study in which mediation analyses were conducted.

IM: Intervention study in which mediation analyses were conducted.

◎: The variable of anticipated affect was significantly, positively, and more strongly associated with the outcome than cognitive variables.

〇: The variable of anticipated affect was significantly and positively associated with the outcome or significantly and positively influenced the outcome.

⊖: The variable of anticipated affect was significantly and negatively associated with the outcome.

●: The variable of anticipated affect was not significantly associated with the outcome or did not significantly influence the outcome.

Three studies investigated the relationship between anticipated regret from inaction and self-reported vaccination behavior using a cross-sectional design. Among these three studies, two studies reported that anticipated regret from inaction was significantly and positively associated with self-reported vaccination behavior [36,37]; one of the two studies showed that anticipated inaction regret was significantly, positively, and more strongly associated with vaccination behavior than cognitive beliefs [36]; in contrast, another study reported no significant association [38]. One cross-sectional study reported that anticipated regret from inaction was significantly and negatively associated with vaccine hesitancy [39].

Two cross-sectional studies investigated the relationship between anticipated guilt from inaction and vaccination intention [40,41] and reported that anticipated guilt was significantly and positively associated with intention to receive vaccination.

Two cross-sectional studies investigated the relationship between anticipated regret from action and self-reported vaccination behavior [38,39] and between anticipated regret from action and vaccination intention [28], and reported that anticipated regret from action was significantly and negatively associated with each outcome (i.e., the more strongly participants had anticipated regret from action, the less likely they were to be vaccinated).

Two cross-sectional studies investigated the relationship between anticipated pride from action and vaccination intention [41] and between anticipated positive feelings from action and vaccination intention [31], and reported that each anticipated action positive affect was significantly and positively associated with intention to receive vaccination (i.e., the more strongly individuals anticipated positive affect from action, the higher their vaccination intention). One intervention study reported that anticipated pride from action had a significantly positive impact on intention [35].

## 4 Discussion

### 4.1 State of the art and gaps in the literature

We reviewed studies that investigated the anticipated affect associated with COVID-19 vaccination. Regarding the types of anticipated affect, this review showed that most studies (14 studies) focused anticipated regret from inaction (i.e., not vaccinated). This result was consistent with previous studies [17]. In contrast, only two studies examined guilt from inaction. Because people who infect novel coronavirus with significant others without being vaccinated may feel guilt; hence, the anticipated guilt from inaction may encourage them to get COVID-19 vaccines. We should investigate the effects of anticipated guilt from inaction on COVID-19 vaccination in the future.

Only three studies examined anticipated positive affect from action (i.e., vaccination), such as pride. Previous studies have often focused on fear appeals using the protection motivation theory and an extended parallel process model [42]. However, the world health organization recommends “appealing to people rather than blaming, scaring, or threatening them” as a countermeasure to COVID-19 pandemic fatigue [43]. People have negative affect such as regretas well as positive affect such as pride [16]. Studies to promoted COVID-19 vaccination should consider both positive and negative affects. Future studies should further investigate the effect of positive affect.

This review showed that only three studies investigated anticipated regret from action (i.e., vaccination). Because the COVID-19 vaccines were rapidly developed, people perceived low safety and efficacy of the COVID-19 vaccines and that perception was a barrier to COVID-19 vaccination [[44], [45], [46], [47]]. One qualitative study included in the present review reported that mothers felt anticipated guilt regardless of whether their children received the COVID-19 vaccine [48]. Considering these factors, anticipated negative affect from action could be a barrier to COVID-19 vaccination. Future studies should investigate the influence of the anticipated negative affect from action on the COVID-19 vaccination, paying attention to urgently developed COVID-19 vaccines.

Regarding study design, this review showed that most quantitative studies used a cross-sectional design. No longitudinal study was conducted; this may be because it has not been long since the COVID-19 vaccination program began. Only one study conducted an intervention; this could be because it is difficult to experimentally change how much regret or guilt an individual anticipates [15]. However, researchers can manipulate participants’ anticipated affect by asking them about their anticipated affect [24]. Previous studies investigated this question-behavior effect for recommending condom use [49] and cervical cancer screening [50]. We studies should investigate the impact of anticipated affect on COVID-19 vaccination in intervetion designs in the future.

Regarding the outcomes, this review showed that most studies investigated vaccination intentions. Only a few studies investigated vaccination behaviors. Moreover, the examined behaviors were self-reported by participants; no study examined the actual vaccine uptake. Because the COVID-19 vaccination program began in December 2020 on an emergency basis while the implementation system was being built, it may have been difficult to track the study participants and obtain data on vaccination history. However, considering that the administrative structure of COVID-19 vaccine programs has developed with time, it will now be easier to track everyone's vaccination history. Additionally, the vaccination history of healthcare workers may be easier to track than that of laypersons. For example, a previous longitudinal study on influenza vaccination extracted vaccination data of healthcare workers from a database of hospitals and reported that anticipated inaction regret significantly and positively predicted influenza vaccine uptake [51]. Researchers studies should investigate the vaccination behavior as a primary outcome in the future.

Regarding the mediating model, this review showed that approximately one-third of the studies (five studies) investigated it. One study showed that perceived susceptibility and benefits of vaccination were associated with vaccination intention, which was partially mediated by anticipated guilt from inaction [40]. Another study showed that perceived susceptibility was associated with vaccination behavior mediated by anticipated regret from inaction [36]. These studies suggest that anticipated affect is a more proximal driver of vaccination intention and behavior than cognitive belief. Examining the relationship between cognitive variables, affective variables, and vaccination outcomes will help reveal the psychosocial mechanisms underlying the uptake of COVID-19 vaccination and enable effective interventions to encourage increased rates of vaccination. Researchers should build on the findings on the relationship between cognitive and affective variables and COVID-19 vaccination outcomes in the future.

### 4.2 Implications for future studies and practices

This review explored affective influence versus cognitive influence on COVID-19 vaccination outcomes. Most included studies showed that anticipated regret and guilt from inaction (i.e., not vaccinated) were significantly and positively associated with COVID-19 vaccination outcomes. Furthermore, three studies reported that anticipated regret from inaction was significantly, positively, and more strongly associated with vaccination outcomes than cognitive beliefs, such as perceived susceptibility and severity, social norms, subjective norms, perceived behavioral control, and perceived barriers [27,32,36]. Previous longitudinal studies showed that anticipated regret from inaction predicted influenza vaccination [[51], [52], [53], [54]], HPV vaccination [55,56], and childhood vaccination behaviors [57,58]. Although the included studies in this review were observational studies, the results indicate that the more strongly the people anticipated regret from inaction, the more likely they were to get COVID-19 vaccines. However, one intervention study reported that a message targeting anticipated inaction regret did not significantly increase vaccination intention, whereas a message targeting anticipated action pride did [35]. Further interventional studies should be conducted to examine the effect of targeting anticipated negative affect from inaction, such as regret and guilt, on encouraging the COVID-19 vaccination.

Three cross-sectional studies showed that the more strongly individuals anticipated regret from action (i.e., vaccinated) regarding adverse events, the lower their vaccination intention [28,38,39]. As mentioned earlier, concerns about the safety of urgently developed COVID-19 vaccines are barriers to vaccination [[44], [45], [46], [47]]. Researchers should examine the effect of lowering anticipated action regret to encourage COVID-19 vaccination in the future.

Although only three studies examined anticipated positive affect from action (i.e., vaccinated), all those studies reported significant positive associations with [31,41] and a significant positive effect on [35] the COVID-19 vaccination intention. In particular, a randomized controlled study reported that adding a message targeting anticipated action pride about protecting oneself and others to a standard message targeting cognitive beliefs about vaccine safety and efficacy increased COVID-19 vaccination intention more than a standard message targeting cognitive beliefs alone [35]. Previous studies have reported that the belief that vaccines can stop the pandemic is a factor that encourages the uptake of COVID-19 vaccination [59]. Accordingly, anticipated positive affect from action, such as pride in receiving vaccination to protect others and stop the spread of the pandemic, may help promote the COVID-19 vaccination.

Additionally, government agencies and health professionals may also be able to appeal to other types of anticipated positive affect from action, such as joy, to encourage COVID-19 vaccination (“Let's get COVID-19 vaccination certificate to enjoy leisure activities”). Future studies should explore the potential of anticipated positive affect from action in encouraging COVID-19 vaccination.

In the second year since the COVID-19 vaccination began, health professionals should pay attention to message fatigue [60,61]. In fact, one study in the United States showed that individuals were already sick of hearing about recommendations of prevention against COVID-19 and related statistics in the first year of COVID-19 pandemic [60]. Another study showed that the stronger the message fatigue toward information on COVID-19, the lower their adherence to infection prevention behaviors [62]. Researchers who have examined message fatigue recommend to increase the repertoire of messages that are different from the standard messages [63]. The results of this review indicate that health communication practitioners may be able to use not only messages appealing to cognitive beliefs but also messages targeting anticipated affect to increase the repertoire of messages to promote COVID-19 vaccination, which may consequently help reduce message fatigue and sustain message effectiveness.

To encourage the uptake of the COVID-19 vaccination, health professionals can utilize messages targeting anticipated negative affect from inaction, such as regret and guilt (e.g., “If you do not get COVID-19 vaccines, you may feel regret or guilt when you develop COVID-19 and infect your loved ones with the virus. Choose to get the COVID-19 vaccines to avoid feeling regret or guilt”), and anticipated positive affect from action, such as pride (e.g., “Feel pride in protecting others and contribute to stopping the pandemic by getting COVID-19 vaccines”) as well as messages appealing to cognitive beliefs (e.g., addressing perceived vaccine efficacy and safety).

Several limitations of this scoping review should be acknowledged. Although we adopted a comprehensive search strategy, relevant studies may have been missed. The present review did not include literature written in languages other than English. This review did not assess risk of bias. This review did not conduct a quantitative meta-analysis.

## 5 Conclusions

Most studies indicated a relationship between anticipated negative affect (e.g., regret, guilt) from not receiving vaccination and intention to get COVID-19 vaccines; namely, the more strongly individuals anticipated negative affect from inaction, the more likely they were to get COVID-19 vaccines. Additionally, a few studies showed a relationship between anticipated positive affect from action (i.e., vaccinated), such as pride, and intention to get COVID-19 vaccines; namely, the more strongly individuals anticipated positive affect from action, the more likely they were to get COVID-19 vaccines. In contrast, a few studies showed a negative relationship between anticipated regret from action due to adverse reactions and vaccination outcomes; namely, the more strongly the participants had anticipated regret from action, the less likely they were to get COVID-19 vaccines. Thus, anticipated affect is related with COVID-19 vaccination outcomes and may have impact on vaccination behaviors. However, most studies were observational studies and adopted intention to receive vaccination as an outcome. More intervention studies should be conducted to investigate the impact of anticipated affect on the uptake of COVID-19 vaccines. Future studies should focus on not only anticipated negative affect from inaction but also anticipated positive affect from action.

## Ethical clearance

The study was granted an exemption from requiring ethics approval by the ethical review committee at Graduate School of Medicine, The University of Tokyo.

## Funding

This work was supported by the 10.13039/501100001691Japan Society for the Promotion of Science KAKENHI (20K10397).

## Data availability statement

No data was used for the research described in the article. No data associated with this study has been deposited into a publicly available repository.

## Additional information

No additional information is available for this paper.

## CRediT authorship contribution statement

**Tsuyoshi Okuhara:** Writing – review & editing, Writing – original draft, Methodology, Investigation, Formal analysis, Data curation, Conceptualization. **Ritsuko Shirabe:** Writing – review & editing, Data curation. **Yumi Kagawa:** Writing – review & editing, Data curation. **Hiroko Okada:** Writing – review & editing, Funding acquisition. **Takahiro Kiuchi:** Writing – review & editing, Supervision.

## Declaration of competing interest

The authors declare that they have no known competing financial interests or personal relationships that could have appeared to influence the work reported in this paper.
