# Supplementary material for: Encouraging COVID-19 vaccination by focusing on anticipated affect: A scoping review
Source: Heliyon. 2023 Nov 18;9(12):e22655. doi: 10.1016/j.heliyon.2023.e22655 (PMC10709050; doi:10.1016/j.heliyon.2023.e22655)
Supplement: Multimedia component 1 [file mmc1.docx]

**Appendix 1. The combinations of keywords used for the literature search.**

(anticipated OR anticipatory) AND (affect OR affective OR emotion OR regret OR guilt OR worry OR fear OR disgust OR embarrassment OR pride OR satisfaction) AND (vaccines OR vaccinations OR immunizations OR vaccine hesitancy OR vaccine refusal OR vaccine reluctance OR vaccine confidence OR vaccine willingness OR vaccine acceptance OR vaccination hesitancy OR vaccination refusal OR vaccination reluctance OR vaccination confidence OR vaccination willingness OR vaccination acceptance)
